# The *Candida albicans* Biofilm Matrix: Composition, Structure and Function

**DOI:** 10.3390/jof3010014

**Published:** 2017-03-08

**Authors:** Christopher G. Pierce, Taissa Vila, Jesus A. Romo, Daniel Montelongo-Jauregui, Gina Wall, Anand Ramasubramanian, Jose L. Lopez-Ribot

**Affiliations:** 1 Department of Biology, University of the Incarnate Word, San Antonio, TX 78209, USA; cpierce@uiwtx.edu; 2 Department of Biology and South Texas Center for Emerging Infectious Diseases, The University of Texas at San Antonio, San Antonio, TX 78294, USA; taissa.Vila@utsa.edu (T.V.); jesus.romo@utsa.edu (J.A.R.); danmon45@gmail.com (D.M.-J.); gina.wall@utsa.edu (G.W.); 3 Department of Biomedical, Chemical & Materials Engineering, San José State University, San José, CA 95192, USA; anand.ramasubramanian@sjsu.edu

**Keywords:** *Candida albicans*, candidiasis, biofilms, extracellular matrix, antifungal resistance

## Abstract

A majority of infections caused by *Candida albicans*—the most frequent fungal pathogen—are associated with biofilm formation. A salient feature of *C. albicans* biofilms is the presence of the biofilm matrix. This matrix is composed of exopolymeric materials secreted by sessile cells within the biofilm, in which all classes of macromolecules are represented, and provides protection against environmental challenges. In this review, we summarize the knowledge accumulated during the last two decades on the composition, structure, and function of the *C. albicans* biofilm matrix. Knowledge of the matrix components, its structure, and function will help pave the way to novel strategies to combat *C. albicans* biofilm infections.

## 1. Introduction

**Figure 1:**
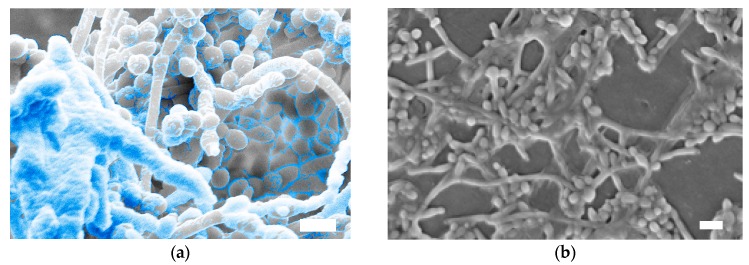
Scanning Electron Microscopy (SEM) images showing the presence of extracellular matrix in *Candida albicans* biofilms. (**a**) Biofilm samples were fixed and dehydrated for processing for SEM; (**b**) biofilm samples were air-dried and not fixed to maximize the preservation of exopolymeric material. The matrix material in **Panel a** has been pseudocolored. Bars are 10 µm.

The ability to form biofilms represents one of the major virulence factors in *C. albicans*, the main etiological agent of candidiasis and now the third-to-fourth most frequent infection in US hospitals [1,2,3]. Biofilms display intrinsic levels of resistance against a majority of antifungal agents [4]. As such, biofilm formation is a major contributor to the unacceptably high mortality rates associated with these infections [1,2]. Sessile cells within these biofilms are embedded within an extracellular matrix (Figure 1) composed of a conglomeration of self-produced exopolymeric materials that envelops the entire structure and confers protection against a variety of environmental insults [5]. Indeed, the presence of this extracellular matrix represents the defining feature of the biofilm life-style. Because of the importance of the matrix during the biofilm mode of growth, studies during the last two decades have focused on its composition, structure, and biological functions. Here we provide a summary of such studies.

## 2. Composition of the *C. albicans* Biofilm Matrix

### 2.1. Extraction Techniques for the Isolation of the C. albicans Biofilm Matrix

Isolation of the biofilm matrix can be challenging, and it is of critical importance to adapt the extraction procedure to the specific type of biofilm under investigation. In the case of *C. albicans*, pioneering studies by the Douglas group in the early 2000s described a basic extraction procedure using sonication, and provided some elementary chemical analyses of matrix components [6,7]. A similar method for the isolation of the biofilm matrix was later adapted for proteomic analyses [8]. The method has been recently improved and refined by the Andes group and adapted for large-scale production to allow for the recovery of milligram amounts of matrix material required for downstream analyses, which has recently been described in detail [9]. This method involves growing *C. albicans* biofilms in large surface area roller bottles, followed by “gentle” sonication for the extraction and isolation of matrix materials. Further purification by sequential filtration, dialyzation, and lyophilization steps renders a matrix material of sufficient purity to allow for subsequent biochemical, structural, and functional analyses [9]. Importantly, the use of this methodology ensures the lack of cell leakage or cell damage, and using electron microscopy it was demonstrated that it specifically removes matrix and does not extract cell wall components [9,10].

### 2.2. Compositional Analyses of the C. albicans Biofilm Matrix

Matrix composition is dynamic and may be affected by the environment; thus, changes in growth conditions such as growth medium, temperature, pH, etc. may lead to compositional alterations. Collectively, biochemical analyses have revealed the overall composition of the *C. albicans* biofilm matrix, with unique components representing each of the four major macromolecular classes, including polysaccharides, proteins, lipids, and nucleic acids [5]. More recently, more in-depth analyses leading to the identification of the biopolymers in the matrix have been facilitated by the implementation of state-of-the-art instrumentation and powerful proteomics, glycomics, and lipidomics techniques [8,10,11]. To date, this has resulted in a rather comprehensive catalog of individual biofilm matrix components.

#### 2.2.1. Proteins

Proteins account for approximately 55% of the dry weight of the *C. albicans* extracellular matrix—more than previously thought and far exceeding the carbohydrate content on a mass basis [10]. Several proteins and glycoproteins extracted from the biofilm matrix were identified using proteomic techniques (two-dimensional gel electrophoresis and mass spectrometry), and there was a striking similarity between matrix proteins and proteinaceous components present in the liquid supernatants from planktonic cultures [8]. These results seem to suggest that the materials secreted during the somewhat “artificial” growth in the laboratory under planktonic conditions will otherwise end up forming part of the matrix during growth as biofilms, which are the most likely mode of growth during infection. Proteins found in the biofilm matrix included a few predicted to form part of the secretome (mostly glycoproteins), but also many secretion-signal-less proteins, with a predominance of glycolytic enzymes as well as heat shock proteins [8,12,13]. A more recent study using more powerful advanced proteomic techniques resulted in the identification of a total of 565 different proteins in the matrix, representing a total of 458 distinct activities [10]. Confirming the results of the previous study, these included several proteins predicted to be secreted but also many without a secretion signal. This suggests a non-canonical secretion pathway and/or the accumulation of proteins after cell death. A functional ontology analysis indicated that a total of 16 different metabolic pathways were represented, with a preponderance of enzymes involved in carbohydrate and amino acid metabolism, and also some enzymes potentially involved in matrix degradation to promote biofilm dispersal [10,14].

#### 2.2.2. Carbohydrates

Like most bacterial biofilm matrices, polysaccharides represent a major constituent of the *C. albicans* biofilm matrix, accounting for approximately 25% of its dry weight [10]. Moreover, as compared to all other biopolymers, the carbohydrate fraction displays the highest degree of complexity within the *C. albicans* biofilm matrix [10]. Arabinose, mannose, glucose, and xylose constitute the most abundant monosaccharides of the total carbohydrate pool, although their relative abundance varies in high versus low molecular weight fractions. When the different fractions were subjected to analysis by Nuclear Magnetic Resonance (NMR), the presence of three major exopolysaccharides was revealed [10]. These are similar to the main polysaccharide components of the *C. albicans* cell wall, although their relative proportions and structure in the biofilm exopolymeric material is different. The most abundant polysaccharides in the *C. albicans* biofilm matrix (constituting about 87%) are mannans—more specifically, α-1,2 branched and α-1,6 mannans. These mannan polysaccharides are found associated with linear (as opposed to highly-branched found in the cell wall) β-1,6 glucans, constituting about 13% of all carbohydrates in an apparent mannan–glucan complex (MGCx), pointing to a physicochemical interaction between glucan and mannan residues. Furthermore, each MGCx component has structural features different than in the fungal wall; for example, the mannan found in the biofilm matrix exists as a much larger structure (up to 12,000 mannose residues) as compared to the cell wall mannan (approximately 150 residues) [10,12]. Contrary to what was previously thought, β-1,3 glucan—which represents the major cell wall polysaccharide and was previously demonstrated to play a major role in biofilm drug resistance (see below) [15,16,17,18]—comprised only a small portion of the total carbohydrate fraction of the biofilm matrix [10]. Furthermore, electron microscopy indicated that the β-1,3-glucan is sparsely distributed throughout the biofilm matrix [10]. Of note, chitin was not detected in the extracellular matrix [10], further corroborating the compositional differences between the biofilm matrix and the cell wall. 

#### 2.2.3. Lipids

Lipids have also been detected in the *C. albicans* biofilm matrix, where they account for approximately 15% of its dry weight [10]. Use of state of the art lipidomics techniques allowed for the identification of the different types of lipids present in the matrix [10]. The lipid profile associated with the *C. albicans* biofilm matrix includes predominantly glycerolipids (99.5%), with a much smaller proportion of sphingolipids (0.5%). The matrix is enriched in neutral glycerolipids (89.2%), while polar glycerolipids are less abundant (10.4%). Different fatty acids are also present on these lipid fractions, the most abundant being oleic and linoleic acids present in the neutral glycerolipids. Palmitoleic, palmitic, stearic, and myristic acids are present in smaller amounts. Phosphatidylethanolamine was the most abundant class of polar glycerolipids in the matrix. Not surprisingly, ergosterol—the main sterol in fungal cell membranes—seems to be the only sterol detected in the biofilm matrix, albeit at very modest concentrations [10]. In addition, consistent with previous reports [19], small amounts of Prostaglandin E2—a precursor of eicosanoids—are also found in the *C. albicans* biofilm extracellular matrix. 

#### 2.2.4. Nucleic Acids

Extracellular DNA (eDNA) has been identified almost universally as part of the extracellular matrix in biofilms formed by different bacterial species [20]. The same is true for *C. albicans*, where the presence of eDNA has been described in the biofilm matrix under a variety of growing conditions [21], constituting around 5% of the weight of the matrix [10]. Proposed mechanisms implicated in eDNA release into the matrix include cell lysis, quorum sensing, and excretion from DNA-containing vesicles [21]. Recently it was demonstrated that the DNA present in the *C. albicans* biofilm matrix is composed largely of random non-coding sequences [10]. 

## 3. Structure of the *C. albicans* Biofilm Matrix

The different exopolymeric constituents of the *C. albicans* extracellular matrix interact with each other to give rise to the overall matrix architecture, resulting in the formation of a cohesive hydrated three-dimensional polymeric network that is characteristic of the biofilm lifestyle [5,10,22]. Although the exact procedures for the assembly of matrix materials are not completely understood, recent investigations have begun to shed light into this process [5,22]. As mentioned before, studies have demonstrated the presence of mannan–glucan complexes (MGCxs), indicating close interactions between these two matrix exopolysaccharides [10]. A more recent report using a combination of genetic and biochemical approaches added important information on the contributions of mannans and glucans to the matrix structure [22]. It was demonstrated that interference with the synthesis or export of an individual constituent resulted in altered concentrations of the other polysaccharides, indicating that matrix biogenesis requires the coordinated delivery of individual matrix exopolysaccharides [22]. However, it was also observed that mixed biofilms containing mutant strains from the different pathways restored matrix structure (and function) [22]. These observations point to the fact that matrix assembly is coordinated extracellularly, and are strongly suggestive of a community behavior where mutants lacking one polysaccharide can be complemented by neighboring cells lacking a different polysaccharide.

Aside from these interactions between exopolysaccharides, other multicomponent interactions involving the different matrix constituents are also likely to occur. These may include both physicochemical interactions and the entanglement of biopolymers that may further contribute to biofilm stability. For example, studies have demonstrated a predominant role for eDNA as a key component of the *C. albicans* biofilm matrix providing structural integrity required for biofilm maintenance [21]. This indicates an important role for eDNA in the overall process of matrix assembly, possibly as a “connector” between the different matrix constituents [21]. It is also likely that matrix proteins may act as intermediaries of covalent linkages between the different exopolysaccharides, similar to what occurs in the cell wall [12]. Moreover, these multicomponent interactions between the different matrix exopolymers yield emergent properties, leading the authors to postulate that—as a whole—the *C. albicans* biofilm matrix displays “properties of an amalgam” [10].

Of note, the overall structure of the biofilm matrix can also change depending on environmental conditions. For example, the formation of *C. albicans* biofilms under conditions of flow normally results in increased production of biofilm matrix [23,24]. This is a method by which biofilms can increase the strength of their structural matrix in response to the mechanical stresses imposed by shear forces.

## 4. Functions of the *C. albicans* Biofilm Matrix

The biofilm matrix is of critical importance to many aspects associated with the *C. albicans* biofilm mode of growth. The biofilm matrix mediates adhesive and cohesive interactions, providing mechanical stability to the biofilms, controlling cells’ dispersion from the biofilm, and can even act as a digestive system that provides a nutrient source for the consortium of cells. Perhaps most importantly, due to the important clinical repercussions, the *C. albicans* biofilm matrix plays a preponderant role in shielding biofilm cells from environmental insults, representing a physical barrier that protects biofilms cells from the attack by the immune system and from antifungal drug treatment during infection.

### 4.1. Mechanical Stability, Adhesive and Cohesive Interactions

As mentioned above, the overall structure of the biofilm matrix is essential for the mechanical stability of the biofilm; as such, it was recently referred to as the “Fungal Super Glue” [5]. The different exopolymeric components of the matrix interact with each other, and the resulting hydrated polymeric network mediates the cohesive forces critical for biofilm maintenance [10,22]. Some of the matrix components are also involved in the initial colonization via adhesion to abiotic and biotic substrates, as well as in long-term attachment to surfaces [5]. Furthermore, the dissolution of these adhesive and cohesive forces is required for biofilm dispersion [14]. 

### 4.2. Antifungal Drug Resistance

Perhaps the most clinically-relevant function associated with the *C. albicans* biofilm matrix is its role in antifungal drug resistance. Although biofilm resistance is multifactorial [25,26], the protection exerted by the matrix is a major contributor to the high levels of resistance displayed by *C. albicans* biofilms. Early studies by the Douglas group already showed a correlation between matrix abundance and levels of resistance against fluconazole and amphotericin B [6,7]. Results of susceptibility testing experiments using cells disaggregated from the biofilms also indicated a contribution of the matrix to resistance [27]. Subsequently, the Andes group demonstrated a predominant role for β-1,3 glucan in the exopolymeric matrix in the resistance of biofilms to fluconazole—mostly through a mechanism of drug sequestration whereby binding to this matrix component prevents the drug from reaching their cellular targets [15,16,17,18]. A study by Vediyappan et al. revealed a similar effect on amphotericin B resistance [28], and further studies extended these observations to other classes of antifungal agents and also other *Candida* species [16]. Another matrix component with a role in antifungal drug resistance is eDNA. Martins et al. reported that the addition of DNase improves the susceptibility of mature *C. albicans* biofilms against some—but not all—antifungal agents [29]. Although the precise mechanism by which eDNA contributes to drug resistance remains unclear, it may be due to reduced drug penetration.

Depending on the configuration of the mating type locus, *C. albicans* can form two types of biofilms: a “pathogenic” a/α biofilm and a “sexual” a/a or α/α biofilm [30]. The pathogenic biofilms are considered to be impermeable, impenetrable, and drug resistant, whereas sexual biofilms lack these traits. Interestingly, these different properties are mostly linked to differences in the matrix [30].

### 4.3. Protection from the Host Immune System

The extracellular matrix also confers protection to cells and soluble mediators of the host immune system, which constitutes a major impediment to effective treatment. The enveloping matrix may effectively mask cell wall epitopes on the surface of *C. albicans* cells that are important for recognition by the host, contributing to immune evasion. Perhaps the best studied to date are the interactions between *C. albicans* biofilms and neutrophils. Xie et al. reported that mature biofilms were resistant to killing by neutrophils and did not trigger reactive oxygen species (ROS), even though neutrophils retained their viability and functional activation potential. This effect was mediated by matrix glucans acting as a decoy mechanism to prevent neutrophil activation [31]. Most recently, the Nett group reported that the extracellular matrix of *C. albicans* biofilms impairs the formation of neutrophil extracellular traps (NETs) [32]. This impairment was also associated with the suppression of ROS production. The authors claim that lack of NETs contributes to immune evasion and provides a survival advantage to cells within the biofilms as compared to their planktonic counterparts. Additionally, the biofilm matrix is responsible for the impenetrability of *C. albicans* a/α pathogenic biofilms by polymorphonuclear leukocytes [30].

### 4.4. Role of the C. albicans Biofilm Matrix in Mixed Fungal/Bacterial Biofilms

Polymicrobial biofilms are generating increased attention, and data is emerging on some unique properties that the *C. albicans* biofilm matrix plays when the fungus is co-cultured with different bacterial species. For example, it is presumably responsible for the generation of hypoxic microenvironments within the biofilm, allowing for the growth of anaerobic bacteria such as *Clostridium perfringens* and *Bacteroides fragilis* [33]. Most recently, the Jabra-Rizk group demonstrated that in mixed *C. albicans*/*Staphylococcus aureus* biofilms, the *C. albicans* biofilm matrix—particularly the secreted exopolymeric β-1,3 glucan—is responsible for conferring bacterial cells with a high degree of tolerance against antibacterial antibiotics [34]. The effect was mostly due to the *C. albicans* biofilm matrix preventing penetration of the antibiotic drugs [34]. In mixed biofilms, the same *C. albicans* matrix carbohydrate also increases ofloxacin tolerance in *Escherichia coli* [35].

## 5. Conclusions and Future Outlook

Infections caused by *C. albicans* continue to represent a major threat to an increasing number of immune- and medically-compromised patients. The ability of *C. albicans* to form biofilms further complicates treatment of these infections and contributes to the increased mortality rates. The presence of a biofilm matrix is the main defining feature of *C. albicans* biofilms. The matrix, formed by a conglomeration of exopolymeric materials with representation of all the major macromolecular classes, envelops the biofilms and confers protection from the surrounding environment. During infection, this translates into protection from host immune responses and—perhaps most significantly—high levels of resistance against antifungal drugs. As such, the presence of the matrix carries important negative clinical repercussions. Work in the last couple of decades has begun to systematically and comprehensively catalog the different components of the matrix, analyze their assembly and spatial and temporal distribution, and examine their physiological roles. Perhaps the main question is how we can harness this accumulated knowledge to the development of novel, effective, and urgently needed therapies against *C. albicans* biofilm-related infections.
